# Radiosensitive effect of curcumin on thyroid cancer cell death induced by radioiodine-131

**DOI:** 10.2478/intox-2014-0011

**Published:** 2014-11-15

**Authors:** Seyed Jalal Hosseinimehr, Seyed Amir Hossein Hosseini

**Affiliations:** Department of Radiopharmacy, Faculty of Pharmacy, Pharmaceutical Sciences Research Center, Mazandaran University of Medical Sciences, Sari, Iran

**Keywords:** 131-I, curcumin, anti-proliferation, MTT, thyroid cancer cell

## Abstract

Curcumin is a natural product widely consumed by humans. It has many biological properties. In this study, we investigated the radiosensitive effect of curcumin on thyroid cancer cells against cellular toxicity induced by 131-I. Human thyroid cancer and human non-malignant fibroblast cells (HFFF2) were treated with 131-I and/or curcumin at different concentrations (5, 10 and 25 µg/ml) for 48 h. The cell proliferation was measured by determination of the surviving cells by using MTT assay. Our results showed that curcumin increased the killing effect of 131-I on thyroid cancer cells, while it exerted no toxicity on HFFF2 cells. This result shows a promising effect of curcumin on the enhancement of therapeutic effects of 131-I in patients.

## Introduction

Radioiodine-131 (^131^I) has been used as the first line of treatment for hyperthyroidism, Graves’ disease and differentiated thyroid cancer. It has a physical half-life of 8.02 days and emits gamma rays and beta particles (Sawin *et al.*, 1997, Zanzonico, 1997, Robbins *et al.*, 2005). It concentrates in thyroid cells and kills tumor cells, yet it has several side effects such as sialadenitis, gastrointestinal symptoms, xerostomia, temporary bone-marrow suppression and neoplasia (Bushnell *et al.*, 1992, Noaparast *et al.*, 2013). ^131^I may also induce genetic damage and chromosomal instability in normal cells that may result in secondary malignancies (Baugnet-Mahieu *et al.*, 1994, Watanabe *et al.*, 2004, Hosseinimehr *et al.*, 2013). The cytotoxic effect of ^131^I is mainly related to beta particles. Ionizing radiation causes cellular injury mainly by producing reactive oxygen species (ROS). ROS can induce lipid peroxidation and damage to cellular membranes and critical macromolecules such as DNA (Little, 2000, Noaparas *et al.*, 2013). Curcumin is a major component of turmeric, produced from the rhizome of the plant Curcuma *longa* (Chendil *et al.*, 2004). Many studies have indicated that curcumin has strong pharmacological activities such as anti-oxidant, anti-cancer (Kuttan *et al.*, 1985), anti-microbial effects (Negi *et al.*, 1999). Curcumin can scavenge free radicals and protect the cellular macromolecules against oxidative stress (Kalpana *et al.*, 2004, Polasa *et al.*, 2004, Singh *et al.*, 2012). Recently we showed that curcumin protected human lymphocytes against genotoxicity induced by ^131^I and it significantly reduced the DNA damage induced by ^131^I *in vitro* (Shafaghati *et al.*, 2014). Although curcumin exhibited protective effects on chromosome damage induced by ^131^I in normal cells, its effect on thyroid cancer cells during ^131^I treatment is not clear.

The aim of this study was to determine the therapeutic effect of curcumin on cell death induced by ^131^I in thyroid human cancer cells and human non-malignant fibroblast cells *in vitro*.

## Materials and methods

### Cell lines

Human non-malignant skin fibroblasts (HFFF2) and human thyroid cancer (Thr.C1-PI 33) cell line were obtained from the Iranian Pasteur Institute (Tehran). The cells were grown at 37°C and 5% CO_2_ in RPMI 1640 medium supplemented with 10% fetal bovine serum (FBS), penicillin 100 IU/mL, and streptomycin 100 µg/ml, all of which were obtained from Gibco (Invitrogen, USA).

### MTT assay

Thyroid cancer and HFFF2 cells were subjected to cell proliferation assay by using MTT. The MTT colorimetric assay is used for evaluation of cell toxicity. The MTT test is based on the strength of mitochondrial enzymes to decrease MTT (pale yellow) to formazan crystals (dark blue). Owing to their impenetrability through the cell membrane, formazan crystals collect in cells (Ashrafi *et al.*, 2012). Cells (20,000) were seeded in 96-well plates. After 24 h incubation, the cells were treated with various concentrations of curcumin (CM) (5, 10 and 25 µg/ml) and were incubated at 37 °C and 5% CO_2_. After 48 h incubation, 20 µL of MTT (5 mg/mL in phosphate buffer saline) was added to each well, and the cells were incubated for 4 hours. After removal of the medium, dimethyl sulfoxide (DMSO) was used to solubilize the formazan compounds and the cell plates were shaken for 10 minutes. The absorbance of every culture well was read on an ELISA Reader (Bioteck, USA). Cells without any treatment were used as control for comparison of absorbance and cell survival.

### Irradiation protocol

Cells were seeded in 96-well plates. After 24 h incubation, the cells were treated with various concentrations of CM (5, 10 and 25 µg/ml) and incubated at 37°C and 5% CO_2_. After 2h incubation, the diluted solution of ^131^I was added at the dose of 10 µCi (100 µl) to each well and incubated for 48 h. MTT assay was performed according to the above protocol.

### Statistical analysis

Data were presented as mean ± standard deviation (SD) of four experiments. Data were compared and the differences were considered significant if the *p*-value<0.05.

## Results

### Effect of curcumin on cell proliferation in thyroid cancer and HFFF2 cells

**Figure 1:**
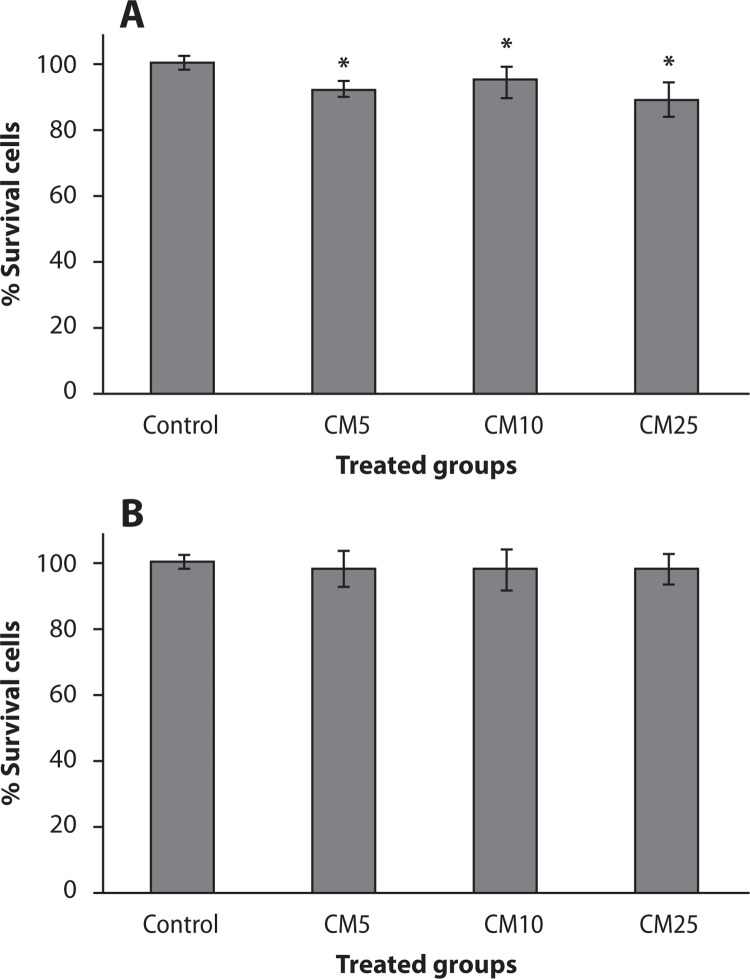
Effect of curcumin (CM) at different concentrations (5, 10 and 25 µg/ml) on thyroid cancer cells (**A**) and non-malignant fibroblast cells (HFFF2) (**B**). Cell proliferation was assayed with MTT test. *p<0.05, comparison CM5, CM10 and CM25 with control

The effect of curcumin on cell proliferation in thyroid cancer and HFFF2 cells is shown in Figure 1. In thyroid cancer cells, a statistically significantly reduced cell proliferation was observed in curcumin treatments at concentrations of 5, 10 and 25 µg/ml (*p<*0.02). The percentage of survival in thyroid cancer cells was 92.5±2.4, 95±4.9 and 89.4±5.3 at concentrations of 5, 10 and 25 µg/ml, respectively. A statistically significant difference was observed between the doses of 5, 10 and 25 µg/ml of curcumin with control for cellular anti-proliferation (Figure 1A). No significant toxicity was observed in HFFF2 cells treated by any of the doses of curcumin (Figure 1B).

### Effect of curcumin and ^131^I combination on cell proliferation in thyroid cancer and HFFF2 cells

**Figure 2:**
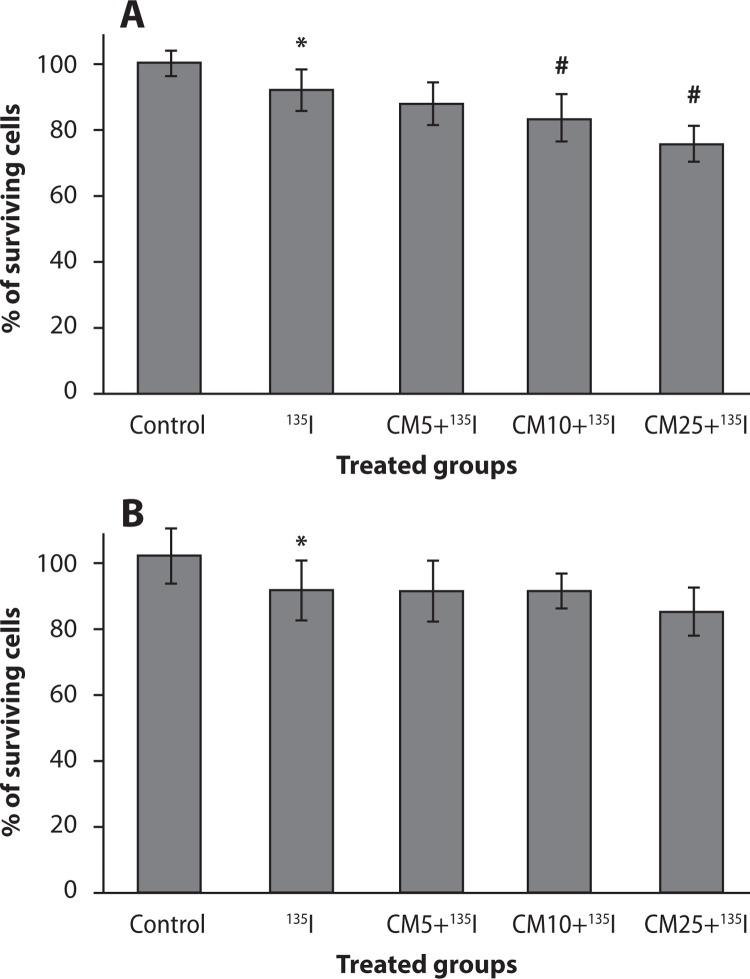
Effect of curcumin (CM) at different concentrations (5, 10 and 25 µg/ml) in combination with ^131^I on thyroid cancer cells (**A**) and non-malignant fibroblast cells (HFFF2) (**B**). Cell proliferation was assayed with MTT test. *p<0.05, comparison control group with ^131^I group; #*p<*0.05, comparison CM10 and CM25 groups with ^131^I group

The combination effects of curcumin and ^131^I on the percentage of cell proliferation in control, curcumin-pretreated, and/or ^131^I treated thyroid cancer and HFFF2 cells are shown in Figure 2. ^131^I significantly reduced the survival rate in thyroid cancer cells by 91%. Thyroid cancer cell proliferation was reduced in pre-treated curcumin groups. Curcumin reduced the percentage of cell survival to 87±6%, 83±7% and 75±5% at concentrations 5, 10 and 25 µg/ml, respectively. Curcumin significantly increased cell death in the dose of 10 and 25 µg/ml in combination with ^131^I as compared to ^131^I alone (*p<*0.05). These results show that curcumin has a synergistic effect with ^131^I on cell growth inhibition in thyroid cancer cells; it is related to the radiosensitive effect of curcumin on thyroid cancer cells treated with ^131^I. Interestingly, curcumin at all doses of 5, 10 and 25 µg/ml did not show any enhancement of toxicity on HFFF2 cells in combination with ^131^I.

## Discussion

In this study, we observed that curcumin exerted a radiosensitive effect on thyroid cancer cells; it reduced significantly cell growth in combination with ^131^I. Curcumin did not exhibit any cellular toxicity in non-malignant fibroblast cells (HFFF2) treated at the same doses with ^131^I. Iodine-131 is widely used for the treatment of thyroid-related diseases. High-dose radioiodine treatment is associated with dose-limited side effects. ^131^I emits gamma and beta rays; the latter ones have a short range board with higher destroying effects on cells as compared to gamma rays. Induction of oxidative stress is one of the main mechanisms for therapeutic and /or side effects of ^131^I. Oxidative stress may cause DNA damage. Several studies showed that curcumin exerted radioprotective effects on normal cells such as human lymphocytes and fibrosis in the rat lung. Protective effects of curcumin are related to free radical scavenging and enhancement of enzymatic and non-enzymatic antioxidants like GSH in cells treated with curcumin (Srinivasan *et al.*, 2006, Cho *et al.*, 2013).

Recently we showed that curcumin significantly protected human lymphocytes from genotoxicity induced by ^131^I. Curcumin reduced micronuclei frequency in lymphocytes in combination with ^131^I (Shafaghati *et al.*, 2014). In this study we tried to evaluate the effect of curcumin on thyroid cancer cell, because it was hypothesized that curcumin could enhance cellular toxicity induced by ^131^I in thyroid cancer cells. Our results showed that curcumin increased radiation toxicity in thyroid cancer cells and it was showed no toxicity on non-malignant human cells induced by ^131^I. These results are promising for using this natural product in combination with ^131^I therapy in patients. Curcumin has been shown to affect mediated several cell signaling pathways such as apoptosis (activation of caspases and down regulation of anti-apoptotic gene products) (Agrawal *et al.*, 2010). Also, curcumin sensitized human cancer cells on exposure to external gamma radiation, which is a dual benefit t of curcumin in patients with cancer therapy (Kunnumakkara *et al.*, 2008, Goel *et al.*, 2010, Lopez-Jornet *et al.*, 2011).

Our findings indicate that curcumin is a promising natural product for patients on radioiodine therapy by radiosensitizing thyroid cancer cells in combination with ^131^I.
